# Identification of Vape Shops in Two North Carolina Counties: An Approach for States without Retailer Licensing

**DOI:** 10.3390/ijerph13111050

**Published:** 2016-10-27

**Authors:** Joseph G. L. Lee, Heather D’Angelo, Jaleel D. Kuteh, Ryan J. Martin

**Affiliations:** 1 Department of Health Education and Promotion, College of Health and Human Performance, East Carolina University, Greenville, NC 27858, USA; kutehj12@students.ecu.edu (J.D.K.); martinry@ecu.edu (R.J.M.); 2 Westat, Rockville, MD 20850, USA; heatherdangelo@westat.com

**Keywords:** tobacco products, commerce, retailers, vaping, e-cigarette

## Abstract

Stores that sell electronic nicotine delivery systems (ENDS) as their primary product are a new phenomenon and often termed “vape shops”. While vape shops are now regulated by state and federal agencies, not all states maintain lists of vape shops in operation. Standard ways of identifying tobacco retailers through off-premise alcohol permits and business listing services may not identify vape shops. We used four online business listing services (i.e., Google Maps, ReferenceUSA, YellowPages.com, Yelp) to identify vape shops in two counties in North Carolina (NC). In one county, we also assessed four vaping web sites. We drove primary and secondary roads to physically validate the identified stores and attempt to identify stores not listed online. To assess the accuracy of the online searches, we calculated sensitivity and positive predictive values (PPVs). This research was conducted in spring and summer 2016 and identified 28 vape shops online. We confirmed 16 vape shops (seven in Pitt County, NC, USA, and nine in Durham County, NC, USA). Online searches ranged in sensitivity, 62.5%–81.3%, and PPVs ranged from 73.3% to 92.3%. Because of the range of sensitivity found among the business listing services, state policymakers should consider uniform licensing requirements for vape and tobacco retailers to more easily track retailers and ensure compliance with regulations.

## 1. Introduction

The use of electronic nicotine delivery systems (ENDS) has been increasing as a replacement for conventional smoking, as a new form of addiction, and as a supplement to cigarette and other tobacco product use [1]. Major tobacco companies as well as independent companies market ENDS [2]. These ENDS products are sold both by conventional retailers who sell tobacco products and a new type of retailer specializing solely in ENDS that does not sell other tobacco products [3,4,5]. Often termed “vape shops”, these brick-and-mortar retailers range from regional chains to small independent retailers, many of whom are run by people who report having been helped in quitting cigarettes through ENDS [3,5]. As of December 2015, there were approximately 10,000 vape shops in the United States [6]. Emerging evidence suggests vape shops may cluster near college campuses [6] and provide information that is not scientifically-based about quitting and harms of ENDS use [3]. Additionally, exposure to marketing at retailers who sell ENDS is associated with youth ENDS use [7,8]. For these reasons, efforts to regulate ENDS products and the practices of ENDS retailers are an important part of promoting population health [9].

On 10 May 2016, the Food and Drug Administration (FDA) established its authority to regulate ENDS with an effective date of 8 August 2016, making ENDS a regulated tobacco product [10]. In doing so, vape shops became subject to FDA restrictions and to the FDA’s retailer inspections program. The FDA’s retailer inspection program has been operating since 2010 (and operated previously from 1997 to 1999) [11]. Since 2010, over 646,000 tobacco retailer inspections have taken place to enforce youth access and marketing provisions of the Family Smoking Prevention and Tobacco Control Act of 2009 [12]. Implementation of the inspections program is subcontracted to states, and states are responsible for creating and maintaining lists of tobacco retailers [13]. To identify vape shops, many states can use licensing lists of tobacco retailers if the tobacco products are defined to include ENDS [14]. Out of the 50 states, Washington, DC, and Puerto Rico, 13 do not have tobacco retailer licensing as of the first quarter of 2016 [15]. For states that do not license or require registration of tobacco or vape retailers, identifying vape shops poses a unique challenge. Tobacco retailers can be identified using business lists of likely tobacco retailers based on North American Industry Classification System (NAICS) codes [16,17] or alcohol permits for off-premise consumption (which identifies convenience stores, grocery stores, and other retailers who are likely to stock tobacco products) [18]. While these approaches would identify many stores that sell ENDS, they would not necessarily identify vape shops that exclusively sell ENDS.

Previous research has examined strategies for identifying vape shops by conducting online searches and verifying results by phone using Amazon Mechanical Turk (mTurk) service crowd sourcing [19]. This approach found that approximately 77% of vape shops on online web sites like Yellowpages.com and Yelp could be verified by crowd-sourced phone calls through mTurk [19]. However, that study did not take into account stores not listed online nor did it assess the sensitivity or positive predictive value (PPV) of each individual data source. Further work is needed to assess if online searches miss vape shops that are not listed and to identify which online sources provide the best sensitivity and PPV. Additionally, little is known about the utility of online search methods for identifying vape shops in rural areas. In rural areas, vape shops have been profiled as important community hubs [20], and past research shows online listings of food retailers in rural areas may perform poorly [21].

Given that inspections of retailers require trained personnel, travel, and are thus expensive, we were interested in the utility of an online search approach to obtain a sampling frame of vape shops that would be practical for state agencies to implement without use of phone verification or mTurk. By using ground truthing (i.e., physically driving roads) to identify the true number of vape shops in our study area, we were able to calculate the sensitivity and PPV of an online search approach to identify vape shops in a state without tobacco retailer licensing.

## 2. Materials and Methods

We used a two-phase search process. After first identifying vape shops online, we then physically ground truthed two North Carolina (NC) counties. We first implemented our search in Pitt County, NC, USA which is located in rural Eastern NC, and home to East Carolina University and a regional medical center. We subsequently implemented our search in Durham County, NC, USA which is located in the more urban research triangle region and is home to Duke University and NC Central University. During ground-truthing, we defined vape shops following Kim et al. [19], as (1) retailers who primarily sell ENDS; (2) to individual customers (i.e., not wholesale); and (3) with a storefront open to the public.

In the Pitt County search, we used four online business listing service sites to identify vape shops. We first conducted online searches in Google Maps, Yelp, and YellowPages following Kim et al. [19], by searching for all combinations of ENDS terminology (“ecig”, “e-cigarette”, “vape”, “vapor”, “vaper”, “vapin”) with each of the 12 cities/towns associated with ZIP codes in Pitt County, NC, USA. We also conducted an online search of ReferenceUSA using NAICS Code 453991 “Tobacco Stores”. There is no NAICS code for vape shops. Two research assistants independently conducted the searching, identification of likely vape shops, and manual de-duplication of results based on name and address from 29 February to 29 March 2016. Search results were nearly identical: one coder identified one more retail location than the other. We used the more comprehensive list for analysis. One author (JGLL) subsequently repeated our search in ReferenceUSA using the newly established Standard Industrial Classification (SIC) code for electronic cigarette retailers (599306) on 28 July 2016; this search resulted in the identification of no additional ENDS retailers who were open at the time of our original search.

In the Durham County search, we repeated the same search protocol but included the SIC code and also searched four vaping web sites (i.e., vapeabout.com, vaporsearchusa.com, vapestores.com, e-cigarette-store-reviews.com). Vaping web sites were included after discussions about our preliminary results from Pitt County indicated researchers were using these sites to identify vape shops. One author (JGLL) conducted online searching for Durham County on 8 August 2016.

We ground truthed Pitt County between 28 March and 1 June 2016, and Durham County between 10 and 16 August 2016. We drove all roads in each county that appear on the 2016 NC State Transportation Map and on the inset maps for the cities of Greenville, NC, and Durham, NC [22]. Previous research has shown that retailers are rarely located in unpopulated areas and tend to cluster with other businesses [23,24]. A search protocol (Supplementary Materials) included driving through strip mall parking lots and walking through malls. Using an iPad Air with an offline Qualtrics Survey application, we recorded the latitude, longitude, posted address, and posted signage of each vape shop identified in our list and sought out any not appearing in the list that could be identified while driving. If a vape shop on our list was not on a road displayed in the NC Transportation Map, we visited it anyway. We again defined vape shops following Kim et al. [19] as: (1) retailers who primarily sell ENDS; (2) to individual customers (i.e., not wholesale); and (3) with a storefront open to the public. As our purpose was to identify operating vape shops, including those not on our online-based list, we did not systematically code possible vape shops for the reason they were ineligible for inclusion.

Once ground truthing was complete, we then calculated the sensitivity and positive predictive value (PPV) of each of the online search sources for the combined counties. Sensitivity calculated the proportion of actual vape shops that were identified in the online search. The value in the numerator for these sensitivity calculations was the number of correctly identified vape shops and the value in the denominator was the number of correctly identified vape shops plus the number of false negatives (which are actual vape shops that were not identified in the online search). Positive predictive value calculated the proportion of vape shops that were identified in the search that were actual vape shops. The value in the numerator for these PPV calculations was the number of correctly identified vape shops and the value in the denominator was the number of correctly identified vape shops plus the number of false positives (which are vape shops identified in the online search that did not meet the vape shop inclusion criteria). East Carolina University’s University and Medical Center Institutional Review Board Office exempted this study from review as not human subjects research (UMCIRB #15-002388).

## 3. Results

Our online search yielded 28 vape shops in the two counties. Ground truthing identified no additional vape shops. That is, no vape shops were identified that were not listed online. Two shops were not located on roads depicted on the NC Transportation Map and would not have been identified by the ground truthing protocol. We physically confirmed (via ground truthing) seven vape shops in Pitt County and nine in Durham County for a total of 16 confirmed vape shops. All 16 of the identified vape shops met the Kim et al. [19], definition of vape shops listed previously. The sensitivity ranged from 62.5 (ReferenceUSA) to 81.3 (Yelp), indicating that Yelp identified more of the existing vape shops than any other data source (Table 1). Positive predictive value ranged from 73.3 (Yellowpages.com) to 92.3 (Google Maps and Yelp), indicating the degree to which if identified as a vape shop, the vape shop was actually a vape shop. Combining Google Maps and Yelp resulted in the identification of all vape shops found through ground truthing. We identified no substantive differences in sensitivity and PPV using the four business listing services between the two counties of interest (data not shown). The four additional online vaping sites used in our Durham County, NC, search performed poorly in regards to sensitivity: vapeabout.com (sensitivity: 55.5, PPV: 100), vaporsearchusa.com (sensitivity: 11.1, PPV: 50.0), vapestores.com (sensitivity: 33.3, PPV: 100), and e-cigarette-store-reviews.com had no stores listed.

## 4. Discussion

Our use of online searches and ground truthing identified vape shops that would likely not be identified by standard ways [16,17,18] of identifying tobacco retailers in states without licensing. Among our online searches, the Yelp web site had the greatest sensitivity in identifying vape shops, identifying 81.3% of existing vape shops. Of the stores identified by Yelp, 92.3% were operating vape shops. Because Yelp content is updated in real time by consumers, it may better reflect currently open vape store locations. The other online sources had only moderate sensitivity for identifying all the vape shops in the study area. These sources may update their existing lists on a specific schedule and may be slower to find new vape shops that are opening or, alternately, their identification strategies may be less sensitive to vape shops. Positive predictive value was high for Google Maps and Yelp (92.3). However, PPV may be less important for state inspections as false negative vape shops that sell tobacco products would still be eligible for inspection as tobacco retailers. States that do not have tobacco retailer licensing should consider the use of Yelp alone or in combination with Google Maps or other online listings of vape shops to complement their identification of tobacco retailers for retailer inspection. However, even the best online source missed nearly one out of five (18.7%) existing vape shops when used alone.

Inspection of vape shops is critical to efforts to ensure that regulations on warning labels, youth access, and childproof packaging are enforced. Vape shop owners appear to be providing health information and advice about quitting smoking to their customers in more in-depth ways than one would encounter at a grocery store, corner store, or convenience store [3]. The information provided by vape shop owners and clerks may not match scientific evidence [25,26,27], and marketing strategies appear to parallel marketing practices used by traditional tobacco retailers [28].

Policy efforts to encourage licensing of vape shops would improve states’ ability to inspect vape shops and enforce regulations. Past research on the effects of licensing shows benefits in promotion of compliance with youth access regulations [29]. In places where vape shop licensing exists, using multiple online sources may be used to check compliance with licensing provisions by identifying businesses that may have opened without obtaining a license. In places considering licensing, this method could generate a list of businesses to which state and local officials can target information about new requirements to current retailers.

While we have focused our discussion of identification of vape shops for inspection purposes, researchers are likely to be increasingly interested in calculating density and proximity of vape shops to examine disparities or correlates of behavior [6,24]. Understanding where and how many vape shops exist in a particular area (for example, near schools) is a critical part of assessing youth exposure to vape shops and ENDS marketing. Use of ENDS has increased dramatically among youth, and nearly 70% of youth were exposed to ENDS marketing in 2014 [30]. Google Maps and Yelp may be helpful for generating a sampling frame with minimal false negatives, but when used alone appear to undercount the true population of vape stores. Using both Google Maps and Yelp together may minimize undercounting of vape shops. In the absence of licensing, multiple sources may be needed for assessing the density of vape stores near schools or parks.

There are important limitations to this study including its geographic area of two counties in a single state. These results may not generalize to other areas of the county. However, the small study area was balanced against a strength of this study: use of ground truthing. This allowed for the physical verification of vape shops in-person as opposed to simply searching online or using phone verification. It is possible that there could have been changes in the vape shop landscape in Pitt County between our online search and the completion of ground truthing, although we are not aware of any misclassification for this reason. Future studies could examine what types of misclassification are present in online searches (e.g., misclassification of tobacco retailers as vape shops, locations out of business, or wholesale locations) and compare misclassification type across online sources. Finally, this study only examined retailers whose primary product is ENDS; ENDS products are available at other retailers not included in this study.

## 5. Conclusions

As vape shops are now selling a product regulated by the FDA, states conducting retailer inspections should consider the use of Yelp and other online sites as a way of identifying vape shops in the absence of statewide licensing requirements. Researchers identifying vape shops from Yelp should be aware that it may miss a meaningful minority of vape shops (e.g., 18.7% of vape shops in our study area). Policy efforts to require licensing or registration of vape shops would improve efforts to inspect vape shops for violations of federal and state regulations.

## Figures and Tables

**Table 1:** Online sources for vape shops, Pitt County and Durham County, NC, USA, 2016.

Measure	Google Maps	ReferenceUSA	Yellowpages	Yelp	Google Maps + Yelp
Sensitivity	75.0	62.5	68.8	81.3	100
PPV **^1^**	92.3	76.9	73.3	92.3	80.0

**^1^** PPV = positive predictive value.

## Supplementary Materials

The following are available online at www.mdpi.com/1660-4601/13/11/1050/s1, Pitt/Durham County Vape Shop Study—Protocol.

## References

[1] Pepper J.K., Brewer N.T. (2014). Electronic nicotine delivery system (electronic cigarette) awareness, use, reactions and beliefs: A systematic review. Tob. Control.

[2] Seidenberg A.B., Jo C.L., Ribisl K.M. (2016). Differences in the design and sale of e-cigarettes by cigarette manufacturers and non-cigarette manufacturers in the USA. Tob. Control.

[3] Cheney M.K., Gowin M., Wann T.F. (2016). Vapor store owner beliefs and messages to customers. Nicotine Tob. Res..

[4] Rose S.W., Barker D.C., D’Angelo H., Khan T., Huang J., Chaloupka F.J., Ribisl K.M. (2014). The availability of electronic cigarettes in U.S. retail outlets, 2012: Results of two national studies. Tob. Control.

[5] Lee Y.O., Kim A.E. (2015). “Vape shops” and “e-cigarette lounges” open across the USA to promote ends. Tob. Control.

[6] Dai H., Hao J. (2016). Geographic density and proximity of vape shops to colleges in the USA. Tob. Control.

[7] Dai H., Hao J. (2016). Exposure to advertisements and susceptibility to electronic cigarette use among youth. J. Adolesc. Health.

[8] Giovenco D.P., Casseus M., Duncan D.T., Coups E.J., Lewis M.J., Delnevo C.D. (2016). Association between electronic cigarette marketing near schools and e-cigarette use among youth. J. Adolesc. Health.

[9] Bhatnagar A., Whitsel L.P., Ribisl K.M., Bullen C., Chaloupka F., Piano M.R., Robertson R.M., McAuley T., Goff D., Benowitz N. (2014). Electronic cigarettes: A policy statement from the american heart association. Circulation.

[10] Food and Drug Administration (2016). Deeming tobacco products to be subject to the federal food, drug, and cosmetic act, as amended by the family smoking prevention and tobacco control act; restrictions on the sale and distribution of tobacco products and required warning statements for tobacco products. Final rule. Fed. Regist..

[11] Clark P.I., Natanblut S.L., Schmitt C.L., Wolters C., Iachan R. (2000). Factors associated with tobacco sales to minors: Lessons learned from the FDA compliance checks. JAMA.

[12] Food and Drug Administration Compliance Check Inspections of Tobacco Product Retailers. http://www.accessdata.fda.gov/scripts/oce/inspections/oce_insp_searching.cfm.

[13] General Services Administration Compliance and Enforcement Tobacco Retail Inspections Program, Solicitation Number: FDA-16-Tobacco-1143096. https://www.fbo.gov/spg/HHS/FDA/DCASC/FDA-16-Tobacco-1143096/listing.html.

[14] Marynak K., Holmes C.B., King B.A., Promoff G., Bunnell R., McAfee T. (2014). State laws prohibiting sales to minors and indoor use of electronic nicotine delivery systems—United States, November 2014. MMWR Morb. Mortal. Wkly. Rep..

[15] Centers for Disease Control and Prevention (CDC) State Tobacco Activities Tracking and Evaluation (State) System: Legislation—Licensure. http://www.cdc.gov/statesystem/.

[16] Rose S.W., Myers A.E., D’Angelo H., Ribisl K.M. (2013). Retailer adherence to family smoking prevention and tobacco control act, North Carolina, 2011. Prev. Chronic Dis..

[17] D’Angelo H., Fleischhacker S., Rose S.W., Ribisl K.M. (2014). Field validation of secondary data sources for enumerating retail tobacco outlets in a state without tobacco outlet licensing. Health Place.

[18] Myers A.E., Hall M.G., Isgett L.F., Ribisl K.M. (2015). A comparison of three policy approaches for tobacco retailer reduction. Prev. Med..

[19] Kim A.E., Loomis B., Rhodes B., Eggers M.E., Liedtke C., Porter L. (2016). Identifying e-cigarette vape stores: Description of an online search methodology. Tob. Control.

[20] Fausset R. Feeling Let Down and Left behind, with Little Hope for Better. http://www.nytimes.com/2016/05/26/us/feeling-let-down-and-left-behind-with-little-hope-for-better.html.

[21] Gustafson A.A., Lewis S., Wilson C., Jilcott-Pitts S. (2012). Validation of food store environment secondary data source and the role of neighborhood deprivation in Appalachia, Kentucky. BMC Public Health.

[22] North Carolina Department of Transportation (2015). North Carolina 2015–2016 Official State Transportation Map.

[23] Caspi C.E., Friebur R. (2016). Modified ground-truthing: An accurate and cost-effective food environment validation method for town and rural areas. Int. J. Behav. Nutr. Phys. Act..

[24] Giovenco D.P., Duncan D.T., Coups E.J., Lewis M.J., Delnevo C.D. (2016). Census tract correlates of vape shop locations in New Jersey. Health Place.

[25] Allem J.P., Unger J.B., Garcia R., Baezconde-Garbanati L., Sussman S. (2015). Tobacco attitudes and behaviors of vape shop retailers in Los Angeles. Am. J. Health Behav..

[26] Basch C.H., Kecojevic A., Menafro A. (2016). Provision of information regarding electronic cigarettes from shop employees in New York City. Public Health.

[27] Nayak P., Kemp C.B., Redmon P. (2016). A qualitative study of vape shop operators’ perceptions of risks and benefits of e-cigarette use and attitude toward their potential regulation by the U.S. Food and Drug Administration, Florida, Georgia, South Carolina, or North Carolina, 2015. Prev. Chronic Dis..

[28] Cheney M., Gowin M., Wann T.F. (2015). Marketing practices of vapor store owners. Am. J. Public Health.

[29] Coxe N., Webber W., Burkhart J., Broderick B., Yeager K., Jones L., Fenstersheib M. (2014). Use of tobacco retail permitting to reduce youth access and exposure to tobacco in Santa Clara County, California. Prev. Med..

[30] Singh T., Marynak K., Arrazola R.A., Cox S., Rolle I.V., King B.A. (2016). Vital signs: Exposure to electronic cigarette advertising among middle school and high school students—United States, 2014. MMWR Morb. Mortal. Wkly. Rep..

