# Supplementary material for: Identification of Vape Shops in Two North Carolina Counties: An Approach for States without Retailer Licensing
Source: Int J Environ Res Public Health. 2016 Oct 27;13(11):1050. doi: 10.3390/ijerph13111050 (PMC5129260; doi:10.3390/ijerph13111050)
Supplement: Supplementary file 1 [file ijerph-13-01050-s001.pdf]

Supplementary Materials: Identification of Vape Shops in Two North Carolina Counties: An Approach for States without Retailer Licensing

Joseph G. L. Lee, Heather D'Angelo, Jaleel D. Kuteh and Ryan J. Martin

Pitt/Durham County Vape Shop Study—Protocol

What happens if I am approached by a staff member of a store?

Politely explain that we are conducting a study of the locations of vape shops in Pitt and Durham County, provide the printed IRB letter, and Dr. Lee's business card.

What happens if I feel there is a safety risk of any type?

Your safety is more important than this research study. Your number one priority is to keep safe. Please keep an eye on your surroundings and immediately leave and go to a safe space if you feel threatened—even if you feel you may feel threatened in the future.

What happens if anything else happens out of the ordinary?

Keep notes about anything that does not quite fit into the data collection and share with Dr. Lee. If you need help or have a question, call:
(252) 328-4661 (Dr. Lee's office)
(XXX) XXX-XXXX (Dr. Lee's cell)
And, of course, call 911 in an emergency.

Study Protocol: Online Search

1. *Use the Spreadsheet Provided to Enter in Findings*

2. *Search Yelp*

   a. For each city with a ZIP code in Pitt County, search the following cities [1].
   b. The cities are: Bellarthur, Bethel, Falkland, Farmville, Fountain, Greenville, Grimesland, Simpson, Stokes, Ayden, Grifton, Winterville.

      i. Each of the following terms: ("ecig", "e-cigarette", "vape", "vapor", "vaper", "vapin")

         1. Read through the results and see if you find any businesses that match our definition of a vape shop: (1) the business *primarily* sells electronic nicotine delivery systems (ENDS) and/or ENDS-related juices/fluids or accessories; (2) the business sells ENDS primarily to individual customers (i.e., is not a wholesale distributor); and (3) the business has a storefront that is open to the public [2].
         2. Enter each location found into the spreadsheet, with the source marked as Yelp under the Vape tab.

3. *Repeat Search Process Using Google Maps*

   a. Note each location, even if it was identified in the Yelp search under the appropriate tabs as from Google Maps.

4. *Search Yellowpages.com*

   a. Note each location, even if it was identified in the Yelp or Google Maps search under the appropriate tabs as from Yellowpages.com.

5. *Search ReferenceUSA*
    a. Log into ReferenceUSA, search U.S. Businesses, select advanced search [3].
    b. Search for: Pitt County, NAICS Code 453991 "Tobacco Stores", and include unverified stores. *Added 2016-08-04:* (Also search SIC Code for E-cigarette shops, 599306.)

6. *Added for Durham County (Bahama, Durham, Research Triangle Park, Rougemont, and Small Portions of Chapel Hill/Morrisville) 4 August 2016:*

7. *Search Vapeabout.com*
    a. Note each location under the Vapeabout.com tab even if it was identified in another search.

8. *Search Vaporsearchusa.com*
    a. Note each location under the Vaporsearchusa.com tab even if it was identified in another search.

9. *Search E-cigarette-store-reviews.com*
    a. Note each location under the E-cigarette-store-reviews.com tab even if it was identified in another search.

10. *Search Vapestores.com*
    a. Note each location under the Vapestores.com tab even if it was identified in another search.

Study Protocol: Field Search ("Ground Truthing")

1. *Ensure That Your iPad is Well Charged and Contains the Commander Compass App as well as the Qualtrics Offline Survey App.*

2. *If Using a New iPad, Update the Commander Compas App to Display the Location in Decimal Degrees.*
    a. First, open the app.
    b. Second, tap the screen to show the preferences icon. Click preferences.
    c. 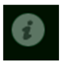 in the lower right corner. Click settings.
    d. Third, click "Geo Coordinates".
    e. 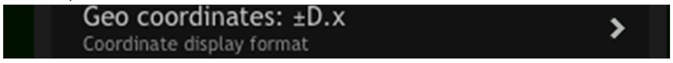

    f. Change it to "±D.x" as shown on the next page. The numbers will change depending on your location.

g. 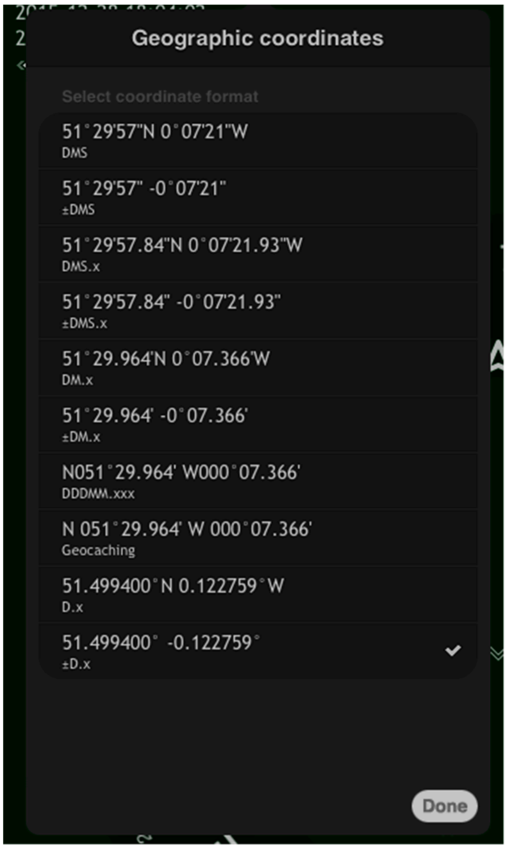

h. Then click done.

3. *Make Sure the Qualtrics App is Loaded and the Survey is Downloaded on the iPad.*

4. *Identify Your Route and Mark It on the Master Map.*

5. *Use the Mileage Log to Mark You Starting Place, Time, and Odometer Reading.*

6. *Drive the Route Looking for Vape Shops. Pull into Strip Malls and Drive through. Park and Walk through Malls (e.g., Greenville mall).*

7. *When You Find A Vape Shop,*

a. If you aren't sure if it's a vape shop, such as for example a hybrid convenience store and vape shop as shown here in Durham:

b. 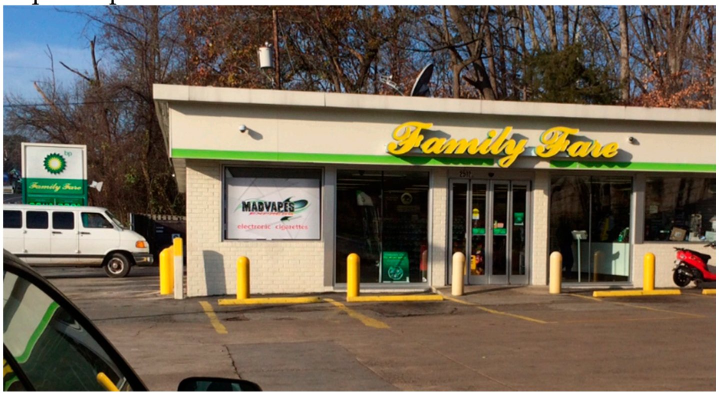

c. Remember that our definition defines vape shop as a place where their primary product for sale is vaping products, electronic cigarettes, etc. So, a mini-vape shop within a convenience store would not meet our definition.

8. *Park near the Vape Shop in A Safe Space.*
    a. Check your surroundings for safety.
    b. If you have any concerns about safety, leave immediately.

9. *Open the Commander GPS App. Give It A Moment to Locate. Then Tap and Hold on the GPS Coordinates in the Upper Right.*
    a. 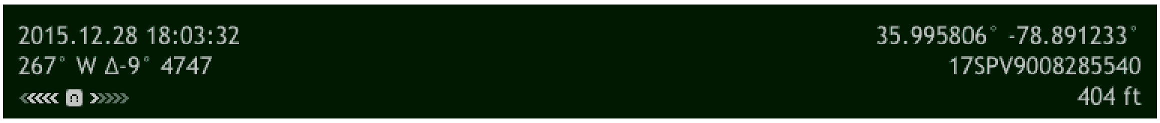

    b. Then click "Copy Location"
    c. 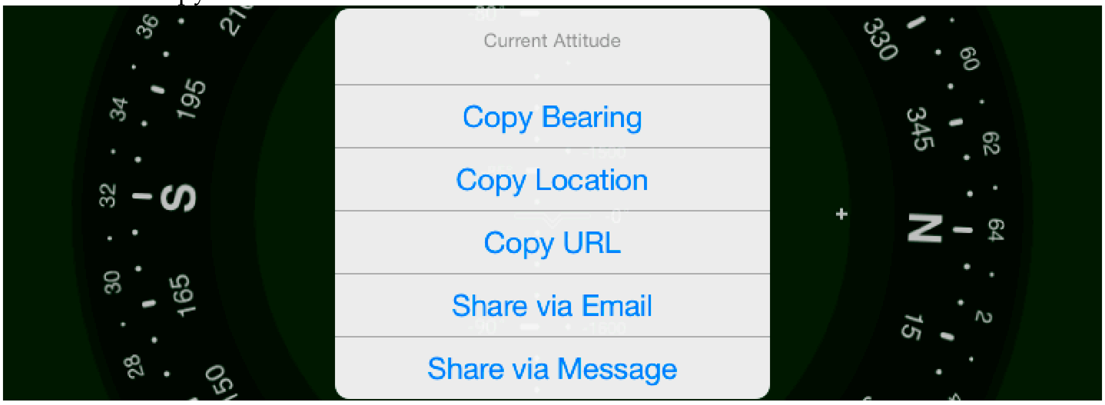

10. *Now Load the Qualtrics App.*

11. *Fill out the Qualtrics Questions.*
    a. Hold down and paste in the location in the location field. Do not edit what is pasted.
    b. If the store is out of business or closed, please fill out the questions anyway.
    c. There is a question about price promotions. We are defining price promotions as signs on the exterior of the store that talk discounts, buy-one-get-one-free, some amount of money off of prices, or say "clearance", "sale", or "discounted".

12. *Mark This as A Stop on Your Mileage Sheet. Continue Driving Your Route.*

Here are examples of Vape shop storefronts:

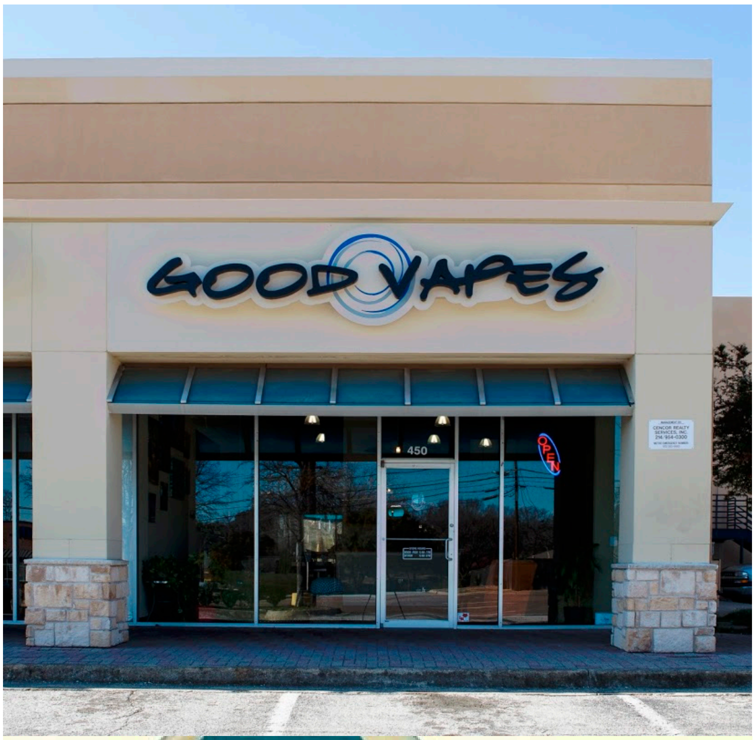
GOOD VAPES
450
OPEN

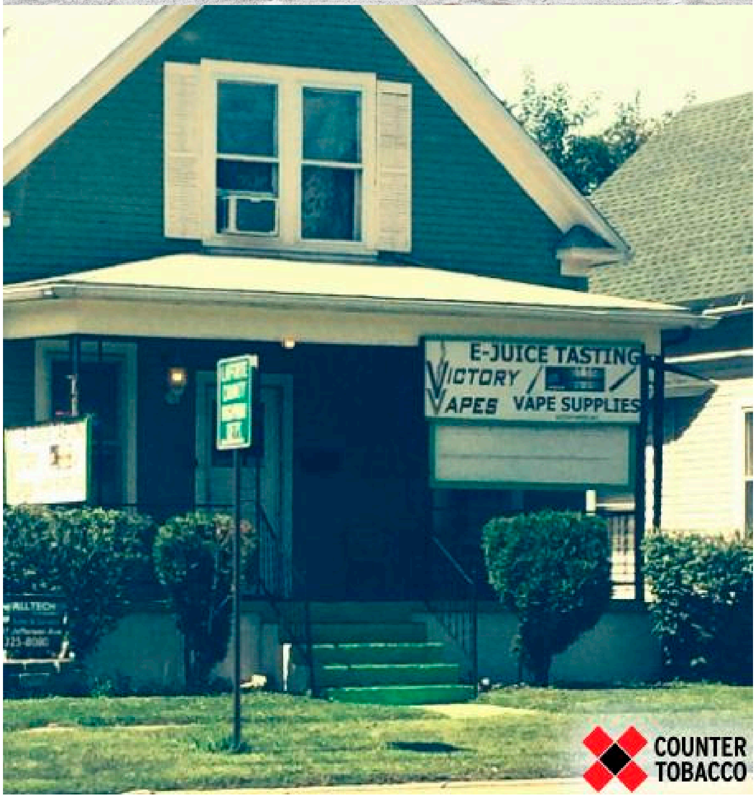
E-JUICE TASTING
VICTORY VAPES
VAPE SUPPLIES
COUNTER TOBACCO

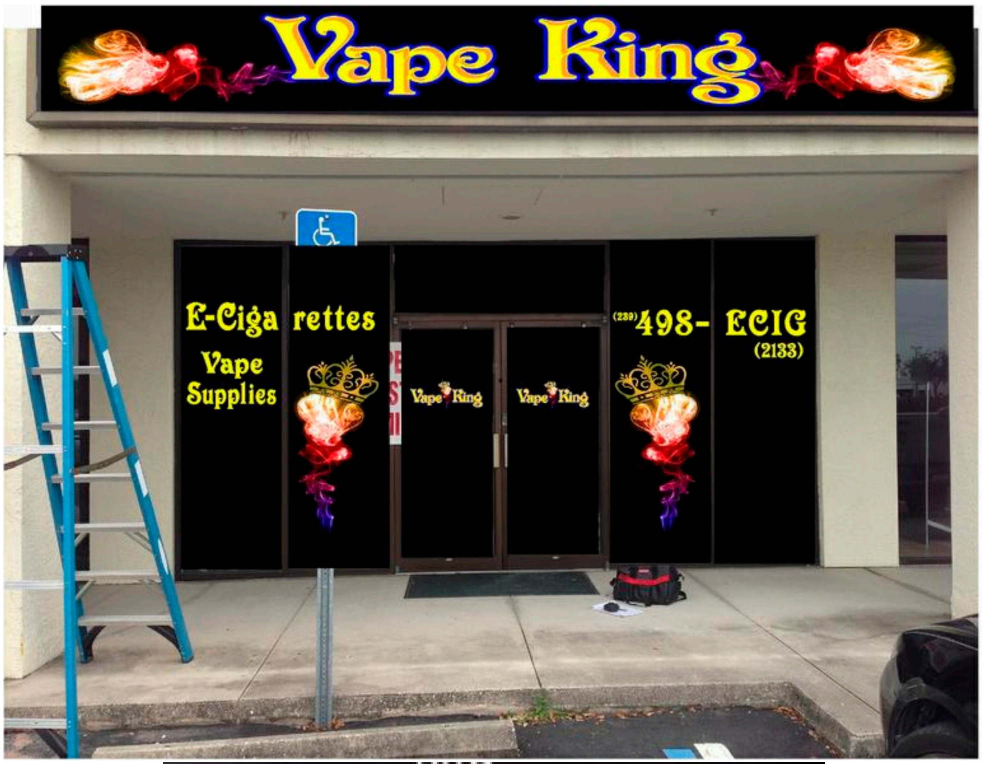

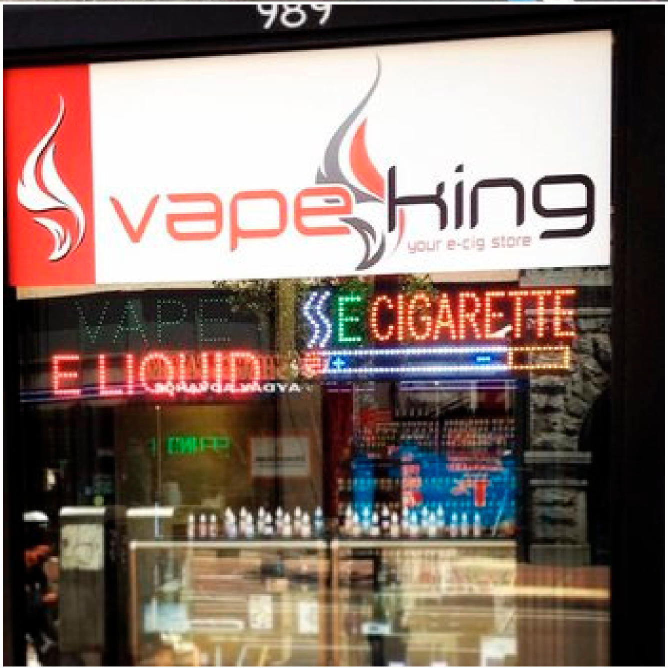

References

1. Datasheer LLC. Pitt County, NC Zip Codes. Available online: https://www.zip-codes.com/county/nc-pitt.asp (accessed on 24 October 2016).
2. Kim, A.E.; Loomis, B.; Rhodes, B.; Eggers, M.E.; Liedtke, C.; Porter, L. Identifying e-cigarette vape stores: Description of an online search methodology. *Tob. Control* **2016**, *25*, e19–e23.
3. ECU Libraries. ReferenceUSA. Available online: https://lib.ecu.edu/databases/view/561 (accessed on 24 October 2016).

© 2016 by the authors; licensee MDPI, Basel, Switzerland. This article is an open access article distributed under the terms and conditions of the Creative Commons by Attribution (CC-BY) license (http://creativecommons.org/licenses/by/4.0/).
